# Appreciative Leadership, Workplace Belongingness, and Affective Commitment of Nurses: The Mediating Role of Job Crafting

**DOI:** 10.1155/2024/2311882

**Published:** 2024-07-05

**Authors:** Manal Saleh Moustafa Saleh, Zaineb Naiem Abd-Elhamid, Nouf Afit Aldhafeeri, Hamad Ghaleb Dailah, Atallah Alenezi, Mohamed Zoromba, Hanan Elsaid Elsabahy

**Affiliations:** ^1^ College of Applied Medical Science Shaqra University, Shaqra, Saudi Arabia; ^2^ Faculty of Nursing Zagazig University, Zagazig, Egypt; ^3^ College of Nursing King Saud bin Abdulaziz University for Health Sciences, Riyadh, Saudi Arabia; ^4^ King Abdullah International Medical Research Center, Riyadh, Saudi Arabia; ^5^ Ministry of the National Guard-Health Affairs, Riyadh, Saudi Arabia; ^6^ College of Nursing Jazan University, Jazan, Saudi Arabia; ^7^ College of Nursing Prince Sattam Bin Abdulaziz University, Al Kharj, Saudi Arabia; ^8^ Faculty of Nursing Mansoura University, Mansoura, Egypt

## Abstract

**Aim:**

This study aimed to investigate the appreciation leadership, workplace belongingness, and affective commitment among nurses, with a specific focus on the mediating role of job crafting.

**Background:**

Leadership, particularly in healthcare care, significantly influences employee experiences and outcomes. Appreciative leadership fosters a positive work environment, valuing and motivating employees. However, its impact on workplace belongingness and affective commitment among nurses requires further exploration. Job crafting, a mechanism in which employees shape their roles to align with their preferences, strengths, and values, can serve as a mediator in the relationship between appreciative leadership and outcomes, such as workplace belongingness and affective commitment. *Subjects and Methods*. A cross-sectional descriptive study was conducted in nurses from two hospitals (Prince Mohammed bin Abdulaziz and Shaqra General Hospital) in Riyadh City, Saudi Arabia. Four standardized scales were used to assess appreciation for leadership, sense of belonging, affective commitment among nurses, and job crafting; 381 nurses were surveyed. AMOS structural equation modeling (SEM) was used to examine the hypothetical model of the study.

**Results:**

APL significantly affects job-crafting behaviors, belonging, and affective commitment among nurses. Furthermore, job-crafting behaviors significantly affect belonging among nurses and commitment.

**Conclusions:**

This indicates that when nurses perceive their leadership positively, their job-crafting behaviors increase, which in turn enhances their sense of belonging at work. Furthermore, these findings indicate that positive leadership perceptions directly improve nurses' commitment to their jobs. This study recommended that educational programs can upgrade leadership styles and change practice levels. *Implications for Nursing Management*. Nursing managers should focus on cultivating appreciative leadership behaviors, such as providing regular feedback, recognizing achievements, and fostering a supportive work culture. Organizations can encourage the creation of jobs among nurses by offering opportunities for autonomy, skill development, and flexibility in job roles.

## 1. Introduction

In contemporary organizational settings, the role of leadership in shaping employee experiences and outcomes has received significant attention. Leadership becomes critical in healthcare settings, as nurse performance and well-being directly affect patient care. Appreciative leadership stands out among leadership philosophies in that it places a strong focus on identifying and developing the potential and strengths of individuals to create a positive work environment [1, 2].

Appreciative leadership is characterized by its focus on strengths, opportunities, aspirations, and results (SOAR). Encourage leaders to cultivate a culture of appreciation, where employees feel valued, empowered, and motivated to contribute their best efforts [3]. Although the potential benefits of this leadership style have generally been acknowledged, more research is needed to fully understand how it can improve nurses' sense of affective commitment and belonging in the workplace [3, 4].

Workplace belonging refers to the sense of connection, acceptance, and inclusion that individuals experience within their work environment [5]. Affective commitment, on the other hand, reflects an employee's emotional attachment and identification with their organization, leading to greater loyalty and engagement. Both factors are crucial to promote a positive organizational environment and facilitate employee well-being and performance [6].

In addition, the concept of job creation has emerged as a mechanism through which employees actively shape their roles and experiences to align with their preferences, strengths, and values. Job crafting involves proactive modification of job tasks, relationships, and perceptions to improve motivation, satisfaction, and performance [7]. Given its relevance to employee autonomy and engagement, the design of the job can serve as a key mediator in the relationship between appreciated leadership and outcomes such as belonging to the workplace and affective commitment [8].

This study aims to investigate the appreciative leadership, workplace belongingness, and affective commitment among nurses, specifically focusing on the mediating role of job crafting. By examining these relationships, insights can be gained into the mechanisms through which appreciative leadership influences nurse outcomes, thereby informing leadership practices and organizational interventions aimed at improving employee well-being and performance in healthcare settings.

In general, this research seeks to contribute to theoretical understanding and practical implications on the impact of leadership on employee experiences and results, with implications for fostering a supportive and engaging work environment in the nursing profession.

### 1.1. Theory and Hypotheses

#### 1.1.1. Appreciative Leadership

The nursing field has faced previously unheard-of difficulties recently, which has highlighted the vital role that capable leadership plays. The pandemic's increased mental and physical demands on nurses resulted in considerable stress and burnout, which raised turnover rates in healthcare settings globally. These findings underline the necessity of leadership strategies that not only meet urgent clinical needs but also cultivate a positive and encouraging work atmosphere [9]. With its focus on identifying and fostering each nurse's unique talents and potential, appreciative leadership stands out as a particularly pertinent strategy. Appreciative leadership can mitigate burnout and strengthen nurses' emotional ties to their organizations by emphasizing positive reinforcement, personal development, and the value of each team member's efforts. This could potentially stabilize workforce dynamics in these challenging times [10].

The benefits of grateful leadership have been highlighted in recent research, especially in the healthcare industry. Research has continuously shown a substantial correlation between improved job satisfaction and decreased workplace stress and appreciative leadership methods, which emphasize employee strengths acknowledgment and empowerment. For example, Janet (2017) observed that patient care outcomes and nurse retention were significantly improved in hospital settings when appreciative leadership was implemented [11]. In a similar vein, the authors in [12] found that healthcare personnel who used appreciative leadership demonstrated higher levels of creativity and cooperation. These results highlight how grateful leadership can improve operational efficiency and create a favorable organizational climate in healthcare settings.

Notwithstanding these encouraging signs, there are still large gaps in our knowledge of the precise ways in which appreciating leadership affects important psychological outcomes like affective commitment and workplace belongingness in nurses. The majority of previous research has concentrated on work satisfaction and retention in general, paying less attention to the more profound emotional and psychological processes that mediate these associations [13]. Moreover, not enough attention has been paid to the function of job crafting as a potential mediator in the dynamics between appreciating leadership and nursing outcomes. This vacuum in the literature points to the urgent need for targeted studies that explore these connections while also elucidating the mechanisms by which appreciating leadership can bring about change in the nursing setting [14, 15]. By addressing these gaps, our study aims to provide a more comprehensive understanding of how appreciative leadership can be strategically applied to improve both the well-being of nurses and the overall quality of healthcare delivery.

#### 1.1.2. Workplace Belongingness

Workplace belongingness, the sense of acceptance and inclusion within an organization, is crucial for employee well-being, performance, and job satisfaction among healthcare professionals, particularly nurses [16] argue that a sense of belonging is derived from interpersonal interactions between employees at work, which is necessary for a relational value to be a part of that system. Another way to describe belongingness to the workplace is the extent to which a person feels personally acknowledged, respected, included, and supported by others at work. The idea of belonging holds that when one feels that justice has been done, one feels extremely intimate and connected to the other members of the group. According to [17], concerns about justice support moral and logical reasons that result from three fundamental needs: a need for control, a need for purpose in life, and a desire for a sense of belonging.

#### 1.1.3. Affective Commitment

An emotional bond with the organization defines the affective commitment feature of organizational commitment. According to this hypothesis, which has its roots in organizational behavior research, nurses who have a strong emotional bond with their organization are more likely to demonstrate traits like loyalty, initiative, and corporate citizenship. Affective commitment is impacted by things like job happiness, leadership efficacy, and perceived organizational support [18]. An employee's affective commitment is his or her emotional attachment to the organization for which he or she works. Strong affective commitment among staff members makes them more dedicated, engaged, and motivated to achieve organizational goals. Organizational management methods often have a direct impact on this kind of dedication [19].

Sometimes affective commitment is referred to as an affective attachment to an organization characterized by shared values, a desire to remain in the organization, and a willingness to exert effort on its behalf [20]. Workers with affective attachment tendencies are more likely to have a sense of identification and belonging, which increases their involvement with work, motivation to work toward the organization's objectives, and desire to stay with the organization. For this reason, the affective dimension appears to be significant. As a result, the authors in [21] saw commitment as a discriminant between dedication and loyalty. Affective commitment is recognized as a more powerful and reliable measure of organizational commitment than normative or continuous commitment.

#### 1.1.4. Job Crafting

Nurses' work is frequently subjected to psychological stress; therefore, it is essential to create a welcoming work environment and implement administrative regulations that improve nurses' combination with their workplace and promote job crafting [22]. The proactive practice of “job crafting” describes how nurses modify job standards and resources to better suit their skill sets and preferences. The job-creation model identifies three dimensions: growing or focusing on social networks that boost structural resources, lowering onerous job demands, and raising demanding job expectations. Nurses manage their careers by modifying the level of demands and resources associated with their jobs [23]. In the same way, job crafting is divided into three subfactors: task crafting, which involves changing job tasks; relational crafting, which involves changing the dynamics of interactions at work; and cognitive crafting, which involves changing how employees view the importance and meaning of their jobs [24].

  H1: Appreciative leadership positively influences the sense of belonging of nurses.
  Fostering a sense of belonging requires a team to have trust and more social exchanges, which are both improved by appreciative leadership. Leaders who emphasize talents and contributions make nurses feel more important and a part of the team [25]. Research has indicated that emphasizing positive feedback and recognition in leadership greatly enhances staff members' sense of community and belonging [26].
  H2: Appreciative leadership positively influences affective commitment among nurses.
  Commitment theory states that employees' emotional attachment to the organization is strengthened by leadership that shares their beliefs and aspirations, which results in increased affective commitment. Developing strategies and systems that involve and uphold seasoned employees, honor their commitments, and lessen their desire to depart the organization requires strong leadership [27]. Nursing faces burnout due to demanding work, staffing shortages, and hierarchical structures. Appreciative leadership, a relationship-based style, promotes empowerment, encouragement, and positive reinforcement, improving nurses' engagement, work happiness, and commitment to the organization [28, 29].
  H3: Job crafting positively influences nurses' sense of belonging.
  Job crafting allows individuals to reshape their work environment and interactions, thereby enhancing their fit within the organization and increasing their sense of belonging [30]. Research demonstrated that job-crafting activities, particularly those modifying social aspects of work, significantly boost employees' sense of belonging [31].
  H4: Job crafting positively influences affective commitment among nurses.
  Employee loyalty to the organization increases when they can better connect their work with their personal beliefs and interests through job crafting, which makes their work more fulfilling and meaningful. According to a study, when workers participate in job crafting, their affective commitment rises as a result of better job satisfaction and a stronger alignment with the organization's aims [32].
  H5: Job crafting mediates the relationship between appreciative leadership and belonging among nurses.
  By allowing nurses to customize their responsibilities, appreciative leadership may promote job crafting. This increases nurses' sense of belonging as they feel more important and incorporated into the team [30].
  H6: Job-crafting behaviors mediate the relationship between appreciated leadership and affective commitment among nurses, with a significant indirect effect.

The job-crafting behaviors of nurses are probably improved by appreciative leadership, and this has a favorable impact on their affective commitment. This process takes place when nurses experience increased emotional attachment to the company as a result of feeling competent in their tasks and being encouraged by their management [33]. According to a study, work crafting has a crucial mediating function in the relationship between affective commitment and leadership styles, hence bolstering the indirect impact of job crafting on leadership [34].

**Figure 1:**
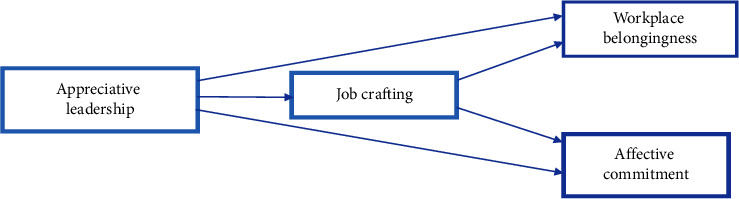
Conceptual model.

Taking into account the seven hypotheses mentioned above, the conceptual research model is presented as follows (Figure 1).

### 1.2. The Study Objective

This study aims to investigate the consequences of appreciative leadership on workplace belongingness and affective commitment among nurses, specifically focusing on the mediating role of job crafting.

## 2. Methodology

### 2.1. Study Design

A cross-sectional descriptive study was selected.

### 2.2. Study Setting

The study was carried out in two hospitals (Prince Mohammed bin Abdulaziz and Shaqra General Hospital) in Riyadh City, Saudi Arabia.

### 2.3. Participants

Because this study is descriptive and the primary outcome is a continuous variable, the sample of nurses was estimated from the addition of the hospital mentioned above, an open-source sample size calculator was used to determine the required sample size by determining the total population size of the previous two hospitals (Prince Mohammed bin Abdulaziz and Shaqra General Hospital). When a population-level outcome factor (p) = 50% ± 5 was hypothesized, and 2000 nurses were studied with confidence limits as % of 100 (absolute +/− %) (d) = 5%, design effect (for cluster surveys—DEFF) = 1, confidence level = 95%, and sample size *n*=[*DEFF∗Np*(1 − *p*)] were calculated. [*∗*(*N* − 1)+*p∗*(1 − *p*)]/[(*d*2/*Z*21 − *α*/2*∗*(*N* − 1)], and 377 was the minimum sample size needed. An additional 30% of nurses needed to compensate for an estimated dropout rate or uncompleted response [35]. The final sample size was 491, and the final sample size recruited for analysis was 381, with a response rate of 77.6%.

A two-phase sampling approach was adopted to enroll nurses as follows:

  Phase 1: Using stratified sampling, the number of participating nurses in each hospital was determined.
  Phase 2: In each hospital, nurses were recruited using convenience sampling. Nurses of licensed staff, those who worked during the study period, and those with at least six months of experience in their present hospital were included. Of the 400 questionnaires distributed, 390 were returned and 9 were invalid. As a result, the final sample size was 381, resulting in a 97.6% effective rate.

### 2.4. Instruments Used in the Study

Data for this study were collected using four standardized scales that were originally developed and used in English.

#### 2.4.1. Appreciative Leadership Scale (ALS)

Appreciative leadership was measured with an 18-item scale developed by [36]. It had been used to find out how nurses felt about their nurse leader's appreciation. There are six elements on this scale for each of the three dimensions listed below: inclusive inquiry, inspiring illumination, and integrity. The internal consistency reliabilities for each subscale according to the early subscale were 0.889, 0.850, and 0.899. A five-point Likert scale was used to rate each item on the scale (1 = all not so to 5 = very different from), where a higher score indicated a higher level of nurses' perception of their nurse leader's appreciation.

#### 2.4.2. Job Craft Questionnaire (JCQ)

Reference [37] created the JCQ to assess the particular types of activities that represented the craft of the job among nurses, which was evaluated using a 15-item scale comprising five items for each of the three dimensions: task creation, cognitive creation, and relational creation. Participants indicate the frequency with which they have participated in each job-crafting activity from 1 (hardly ever) to 6 (very often). According to Slemp and Vella-Brodrick, the reliability scores for task, relational, cognitive, and overall job crafting were, in order, 0.87, 0.89, 0.83, and 0.91.

#### 2.4.3. Workplace Belongingness Scale (WBS)

The belongingness of the workplace among nurses to their job and organization was measured using the 12-item scale developed by [38]. All elements of the instrument were positively formulated, and the nurses' responses were rated on a 5-point Likert scale ranging from 1 (strongly disagree) to 5 (strongly agree), where higher scores represent a greater degree of belonging in the workplace.

#### 2.4.4. Affective Organizational Commitment Scale (AOCS)

Reference [39] created this six-item measure to assess nurses' affective organizational commitment levels. Higher scores indicate a higher level of affective organizational commitment among nurses. The nurses' responses were scored on a five-point Likert scale, with 5 representing strong agreement and 1 representing strong disagreement.

#### 2.4.5. Demographic Information of Study Participants

The demographic information of the participants was collected, including their age, sex, education, marital status, years of nursing experience, current unit, and hospital.

### 2.5. Pilot Study

A preliminary investigation was carried out to assess the clarity of the questionnaires and the time required for their completion. Additionally, a preliminary mean of the outcome variables was calculated to estimate the necessary sample size. The 38 nurses who were selected from participating hospitals for the pilot study were later not included in the final study sample. The questionnaire took 20 to 25 minutes to complete, and the pilot nurses attested to its clarity and understandability. In terms of internal consistency, Cronbach's alpha coefficients were 0.86 for the total appreciation scale for leadership, 0.95 for the total scale for job creation, 0.89 for belonging to the workplace, and 0.90 for the scale of affective organizational commitment, indicating high and acceptable reliability.

### 2.6. Data Collection

Data collection for this study was conducted from February to May 2023, utilizing self-reported assessments from staff nurses. Prior to the commencement of data collection, the necessary ethical approvals were secured. The primary investigator initially met with the chief nurses of each hospital unit to explain the study's objectives and to seek their support in facilitating the data collection process. To enhance the perceived anonymity and reduce potential response bias, nurses meeting the inclusion criteria were provided with sealed envelopes containing the survey questionnaires. These envelopes were distributed in their work areas during their shifts. The cover letter of the questionnaire clearly stated the study's purpose, reassured the participants of the anonymity and voluntary nature of their responses, and instructed them to reflect on their experiences with a specific nurse manager. To further ensure confidentiality and reduce any pressure, nurses were asked to return their completed questionnaires in the sealed envelopes to a designated drop box located in a common area within each unit by a specified deadline. Participation was implied through the voluntary completion and submission of the surveys. This method aimed to minimize any direct interaction with the research team during the submission process, thereby fostering a more confidential and unbiased environment for providing genuine responses.

### 2.7. Ethical Considerations and Consent to Contribute

The Faculty of Nursing Ethics Committee approved ethical approval (No. 21-2-2023). Potential ethical concerns such as participant well-being, privacy, and anonymity were upheld throughout the research procedure. Participants were informed of all study-related materials and their ability to withdraw at any point while the study was in progress. Furthermore, only the authors had access to the sensitive participant data, which was held in a locked, secured cabinet. Lastly, everyone had the guts to raise any pertinent queries.

### 2.8. Statistical Design

AMOS 25 and IBM SPSS 27 were used for data analysis. The study variables and the nursing characteristics were presented using descriptive statistics. Variations in job development, workplace belonging, affective commitment, and appreciation of leadership were examined about sample characteristics using independent *t*-tests and analysis of variance (ANOVA). Pearson's correlation was used to examine the bivariate correlations between the research variables and related subdomains. The proposed model was investigated using AMOS structural equation modeling (SEM). The validity and reliability of the study constructs were evaluated. *p* values with two tails less than 0.05 denote statistical significance.

## 3. Results of the Study

Table 1 shows demographic data samples and differences in study variables. The participants were predominantly between 30 and 35 years of age (39.1%). Most of them (60.6%) were women. Most of them were single (53.3%), 82.7% had a Bachelor's degree in Nursing, and 45.9% had <3 years of experience.

Table 2 analyzes the correlation coefficients among the variables studied, and several significant relationships emerged. In particular, cognitive crafting demonstrated strong positive correlations with relational crafting (*r* = 0.674^*∗∗*^), total job crafting (*r* = 0.902^*∗∗*^), and workplace belonging (*r* = 0.612^*∗∗*^).

Furthermore, affective commitment exhibited a robust positive correlation with workplace belonging (*r* = 0.821^*∗∗*^) and a notable association with total job creation (*r* = 0.690^*∗∗*^). This implies that staff nurses who feel a sense of belonging in their workplace are more likely to be affective, committed to their roles, and involved in crafting their jobs.

The belonging of the workplace demonstrated a remarkably high positive correlation with the integrity subscale of appreciation of leadership (*r* = 0.654^*∗∗*^), suggesting that people who feel like they belong to the workplace are more likely to exhibit integrity in their actions.

Furthermore, inspiring illumination and integrity showed a substantial positive correlation (*r* = 0.509^*∗∗*^). This may imply that nurses who perceive their leaders as inspiring and illuminating also tend to perceive them as having high levels of integrity.

**Figure 2:**
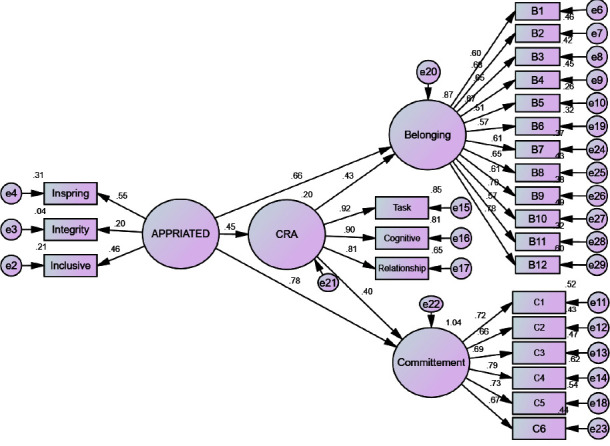
Mediation effect of staff nurses' appreciative leadership among workplace belongingness, affective commitment of staff nurses, and job crafting (*N* = 381).

Table 3 shows the effect of mediation of the appreciation of staff nurses for the leadership between workplace belongingness, affective commitment, and job design, and Figure 2 shows that appreciative leadership significantly affects job-crafting behaviors (*β* = 0.45, *p* < 0.001), workplace belonging (*β* =  0.66, *p* < 0.001) and affective commitment among staff nurses (*β* = 0.78, *p* < 0.001). Furthermore, job-crafting behaviors significantly affected belonging among nurses (*β* = 0.43, *p* < 0.001) and affective commitment (*β* = 0.40, *p* < 0.001). The indirect effect of job-design behaviors on the relationship between appreciative leadership and belonging to the workplace was significant (*β* = 0.19, *p* = 0.001, 95% CI 0.15/0.27). The indirect effect of job-design behaviors on the relationship between appreciated leadership and affective commitment was significant (*β* = 0.18, *p* = 0.002, 95% CI: 0.13/0.24), indicating that job construction behaviors among staff nurses mediated relationships between appreciated leadership, belonging to the workplace, and affective commitment among staff nurses.

## 4. Discussion

To improve nurse well-being and retention, it is critical to comprehend how appreciative leadership, affective commitment, workplace belongingness, and job crafting interact. This knowledge will help build focused treatments and organizational policies [40]. Thus, the purpose of this study is to investigate how job crafting functions as a mediator in the relationship among nurses between appreciative leadership, workplace belongingness, and affective commitment. Organizations can create conditions that support the growth of their nursing staff by clarifying these routes, which will eventually improve patient outcomes and organizational success. Results showed that appreciative leadership has a strong and significant direct effect on workplace belongingness. This finding is valuable because it indicates that nurses' perceptions of their leadership directly contribute to their sense of belonging at work. These results are in line with [41] earlier studies, showing that the direct effect of appreciative leadership on workplace belongingness is significant. Also, the authors in [30] agreed with these findings and reported that appreciative leadership has a positive effect on workplace belongingness. In addition, the findings of the study showed that the effect of appreciative leadership on workplace belongingness is significant [29].

The results of the present study reported that appreciative leadership also has a robust direct effect on affective commitment, indicating that positive leadership perceptions directly enhance nurses' affective commitment to their jobs. These results were supported by [41], who showed that the direct effect of appreciative leadership on affective commitment is significant. Also, the authors in [33] revealed that there is a noteworthy direct correlation between appreciating leadership and affective commitment. Furthermore, according to [42], commitment and appreciative leadership have a favorable relationship with the intentions to turnover. These results are in line with those of [43], who reported that appreciated leadership has a major influence on employee satisfaction and that the relationship between appreciated leadership and affective commitment is accepted.

Related to job-crafting behaviors' effects, the current study showed that job-crafting behaviors have a significant positive effect on workplace belonging, highlighting the importance of these behaviors in fostering a sense of belonging among nurses. These results are consistent with those of [44], who found that job crafting significantly and favorably affects faculty members' sense of belonging at work. Furthermore, this result is in line with a study [41], which discovered a favorable correlation between job crafting and a sense of belonging at work. However, these results are contradictory with [29], who reported that job crafting has an insignificant effect on workplace belongingness.

Furthermore, job-crafting behaviors also positively affect commitment, further establishing the role of job crafting in enhancing organizational commitment. This leads to the same conclusion as [35] who found that highly bonded employees can reduce the severity of stressors and initiate proactive job crafting. He came to the conclusion that job crafting, which raises employee performance, requires a high level of commitment. Also, the authors in [45] it was observed that as organizational commitment rises, job performance increases. When job crafting is added to this relationship, the effect is even higher. This finding is consistent with that of [46], who found a negative relationship between turnover intention and organizational commitment. These results suggest that allowing workers to engage in job-crafting behavior may lessen the intention of turnover, which will eventually increase employee engagement and satisfaction. Furthermore, [47, 48] results showed a moderated effect of affective commitment on the job crafting and concluded that those employees with high levels of need commitment independent of their job crafting.

Finally, appreciative leadership significantly affects job-crafting behaviors, workplace belonging, and affective commitment among nurses. This results congruence with [33], who indicated that the results demonstrate that affective commitment and workplace belongingness are positively impacted by appreciative leadership. Job crafting, however, has a favorable impact on affective commitment but a negligible influence on workplace belongingness. The relationship among job crafting, workplace belongingness, and appreciative leadership is significantly mediated by affective commitment. Also, this result agrees with [41]. The results of this study demonstrate the substantial direct impact that job crafting and appreciative leadership have on a workplace's sense of belonging. Additionally, there is a noteworthy direct impact of appreciating leadership and job designing on affective commitment.

## 5. Conclusion and Recommendations

- Leadership perception: Nurses' perceptions of leadership play a crucial role in shaping their work experience, directly influencing their sense of belonging and commitment and indirectly affecting these outcomes through job-designing behaviors.
- Job crafting: Engaging in job-crafting behaviors appears to be a vital mechanism through which nurses can enhance their sense of belonging and commitment in the workplace.
- Positive work environment: Promoting a positive perception of leadership and encouraging job-building behaviors may lead to a more committed and engaged nursing workforce, which is essential for the well-being of both the staff and the patients they serve.

### 5.1. Implications for Nursing Management

Nurses in the hospital are likely to have high turnover intentions and feelings of disengagement. To ensure higher levels of engagement and lower turnover intentions, management could take the following actions: Nursing managers should focus on cultivating appreciative leadership behaviors, such as providing regular feedback, recognizing achievements, and fostering a supportive work culture. Training programs can be developed to enhance leaders' skills in appreciative communication, conflict resolution, and emotional intelligence. Organizations can encourage the creation of jobs among nurses by offering opportunities for autonomy, skill development, and flexibility in job roles. Policies and practices should align with the principles of appreciation leadership and job crafting, focusing on the importance of employee well-being, engagement, and professional development. These actions are crucial to reducing turnover intentions and disengagement among nurses on the staff.

## Figures and Tables

**Table 1:** Participants' demographics and differences in study variables (*N* *=* 381).

Characteristic	Category	No. (%)	Job crafting		Workplace belonging		Commitment		Appreciative leadership	
			M (SD)	t/F (P)	M (SD)	t/F (P)	M (SD)	t/F (P)	M (SD)	t/F (P)
Age (years)	20: <25	122 (32)	3.36 (0.97)	0.153 (0.858)	3.38 (0.78)	1.45 (0.236)	3.32 (0.81)	2.05 (0.131)	2.67 (0.36)	4.94 (0.008)
	25: <30	110 (28.9)	3.30 (0.85)		3.31 (0.52)		3.41 (0.65)		2.53 (0.35)	
	30: <35	149 (39.1)	3.35 (0.95)		3.45 (0.60)		3.50 (0.71)		2.59 (0.32)	

Gender	Male	150 (39.4)	3.29 (0.87)	0.895 (0.371)	3.27 (0.63)	2.865 (0.004)	3.31 (0.70)	2.46 (0.014)	2.57 (0.32)	1.32 (0.189)
	Female	231 (60.6)	3.37 (0.96)		3.46 (0.64)		3.49 (0.74)		2.62 (0.36)	

Marital status	Single	204 (53.3)	3.23 (0.89)	2.37 (0.018)	3.32 (0.66)	2.177 (0.030)	3.28 (0.82)	4.00 (<0.001)	2.59 (0.38)	0.456 (0.643)
	Married	175 (45.9)	3.45 (0.95)		3.46 (0.62)		3.58 (0.57)		2.61 (0.30)	

Education	BSD	315 (82.7)	3.27 (0.87)	30.7 (<0.001)	3.35 (0.64)	15.08 (<0.001)	3.40 (0.71)	19.72 (<0.001)	2.60 (0.35)	0.55 (0.577)
	Master	33 (8.7)	2.88 (0.86)		3.18 (0.60)		2.97 (0.83)		2.54 (0.38)	
	Technical	33 (8.7)	4.40 (0.76)		3.93 (0.35)		4.03 (0.23)		2.60 (0.29)	

Years in nursing	<3 years	175 (45.9)	3.38 (0.96)	13.56 (<0.001)	3.48 (0.620)	15.19 (<0.001)	3.53 (0.70)	11.77 (<0.001)	2.61 (0.15)	0.237 (0.789)
	3: 6 years	162 (42.5)	3.14 (0.83)		3.20 (0.59)		3.22 (0.73)		2.58 (0.41)	
	>6 years	44 (11.5)	3.92 (0.86)		3.69 (0.72)		3.69 (0.62)		2.61 (0.15)	

Job description	Yes	263 (69)	3.40 (0.89)	1.83 (0.065)	3.54 (0.55)	7.05 (<0.001)	3.60 (0.65)	7.65 (<0.001)	2.64 (0.37)	3.41 (0.001)
	No	118 (31)	3.20 (0.99)		3.04 (0.69)		3.01 (0.73)		2.51 (0.25)	

A Bachelor of Science Degree (BSD). (M) mean. (SD) Standard deviation. (F) One-way analysis of variance. (t) t test for the independent group. (P) significance level.

**Table 2:** Analyzing the correlation coefficients among the studied variables (*N* *=* 381).

Studied variables		Mean (SD)	Cognitive crafting	Relationship crafting	Total job crafting	Workplace belonging	Commitment	Inspiring illumination	Integrity	Inclusive inquiry	Total appreciative leadership
Task crafting	R	3.28 (1.02)	0.848^*∗∗*^	0.749^*∗∗*^	0.948^*∗∗*^	0.555^*∗∗*^	0.603^*∗∗*^	0.221^*∗∗*^	−0.076	0.228^*∗∗*^	0.178^*∗∗*^
Cognitive crafting	R	3.44 (1.04)	1	0.674^*∗∗*^	0.902^*∗∗*^	0.612^*∗∗*^	0.625^*∗∗*^	0.279^*∗∗*^	−0.120^*∗*^	0.157^*∗∗*^	0.160^*∗∗*^
Relationship crafting	R	3.33 (0.99)		1	0.894^*∗∗*^	0.727^*∗∗*^	0.678^*∗∗*^	0.303^*∗∗*^	−0.009	0.328^*∗∗*^	0.287^*∗∗*^
Total job crafting	R	3.34 (0.93)			1	0.690^*∗∗*^	0.694^*∗∗*^	0.290^*∗∗*^	−0.069	0.267^*∗∗*^	0.232^*∗∗*^
Workplace belonging	R	3.39 (0.64)				1	0.821^*∗∗*^	0.394^*∗∗*^	0.025	0.336^*∗∗*^	0.349^*∗∗*^
Commitment	R	3.42 (0.73)					1	0.487^*∗∗*^	0.095	0.378^*∗∗*^	0.440^*∗∗*^
Inspiring illumination	R	2.61 (0.45)						1	0.286^*∗∗*^	0.509^*∗∗*^	0.826^*∗∗*^
Integrity	R	2.53 (0.48)							1	0.301^*∗∗*^	0.654^*∗∗*^
Inclusive inquiry	R	2.63 (0.44)								1	0.793^*∗∗*^
Total appreciative leadership	R	2.60 (0.35)									1

*r* = Pearson correlation. ^*∗*^Correlation is significant at the 0.05 level (2-tailed). ^*∗∗*^Correlation is significant at the 0.01 level (2-tailed). *α* = Cronbach's alpha.

**Table 3:** Mediation effect of staff nurses' appreciative leadership among workplace belongingness, affective commitment, and job crafting (*N* = 381).

Indirect effect	*β*	*p*	BC 95% CI
			Lower/upper
Appreciative leadership on workplace belonging	0.19	0.001	0.15/0.27
Appreciative leadership on affective commitment	0.18	0.002	0.13/0.24

## Data Availability

The data that support the findings of this study are available from the corresponding author upon reasonable request. Also, all data generated or analyzed during this research are included in this manuscript.
